# Switching between internal and external modes: A multiscale learning principle

**DOI:** 10.1162/NETN_a_00024

**Published:** 2017-12-01

**Authors:** Christopher J. Honey, Ehren L. Newman, Anna C. Schapiro

**Affiliations:** Department of Psychological and Brain Sciences, Johns Hopkins University, Baltimore, MD, USA; Department of Psychological and Brain Sciences, Indiana University, Bloomington, IN, USA; Department of Psychiatry, Beth Israel Deaconess Medical Center / Harvard Medical School, Boston, MA, USA

**Keywords:** Acetylcholine, Learning, Timescale, Switching, Contrastive learning, Hippocampus, Sleep, Default mode

## Abstract

Brains construct internal models that support perception, prediction, and action in the external world. Individual circuits within a brain also learn internal models of the local world of input they receive, in order to facilitate efficient and robust representation. How are these internal models learned? We propose that learning is facilitated by continual switching between internally biased and externally biased modes of processing. We review computational evidence that this mode-switching can produce an error signal to drive learning. We then consider empirical evidence for the instantiation of mode-switching in diverse neural systems, ranging from subsecond fluctuations in the hippocampus to wake-sleep alternations across the whole brain. We hypothesize that these internal/external switching processes, which occur at multiple scales, can drive learning at each scale. This framework predicts that (a) slower mode-switching should be associated with learning of more temporally extended input features and (b) disruption of switching should impair the integration of new information with prior information.

## INTRODUCTION

Why do so many of the neural processes supporting memory and perception fluctuate over time? Perceptual thresholds increase and decrease over milliseconds, seconds, and minutes (Palva & Palva, 2011; VanRullen, 2016); the associative strength of hippocampal encoding fluctuates over milliseconds (Hasselmo, 2006; Hasselmo, Bodelón, & Wyble, 2002) and seconds (Douchamps, Jeewajee, Blundell, Burgess, & Lever, 2013; Duncan, Sadanand, & Davachi 2012); and overall arousal fluctuates within the waking state (McGinley et al., 2015b) as well as on the timescale of wake and sleep.

It is possible that these fluctuations are epiphenomena, reflecting arbitrary biological constraints or noise. But there is a cost to breaking from the world: a reduced sensitivity to what is occurring in the environment. Why would the brain be willing to pay this price? We propose that many of these fluctuating processes, across wide-ranging scales and systems of the mammalian brain, can be understood as mode-switching processes that facilitate learning. In particular, we hypothesize that mode-switching enables individual neural systems to construct and refine internal models of the afferent signals that constitute their world. These “models” of afferent signals include any kind of representation that allows a system to improve its predictions about its environment.

**Figure 1.**
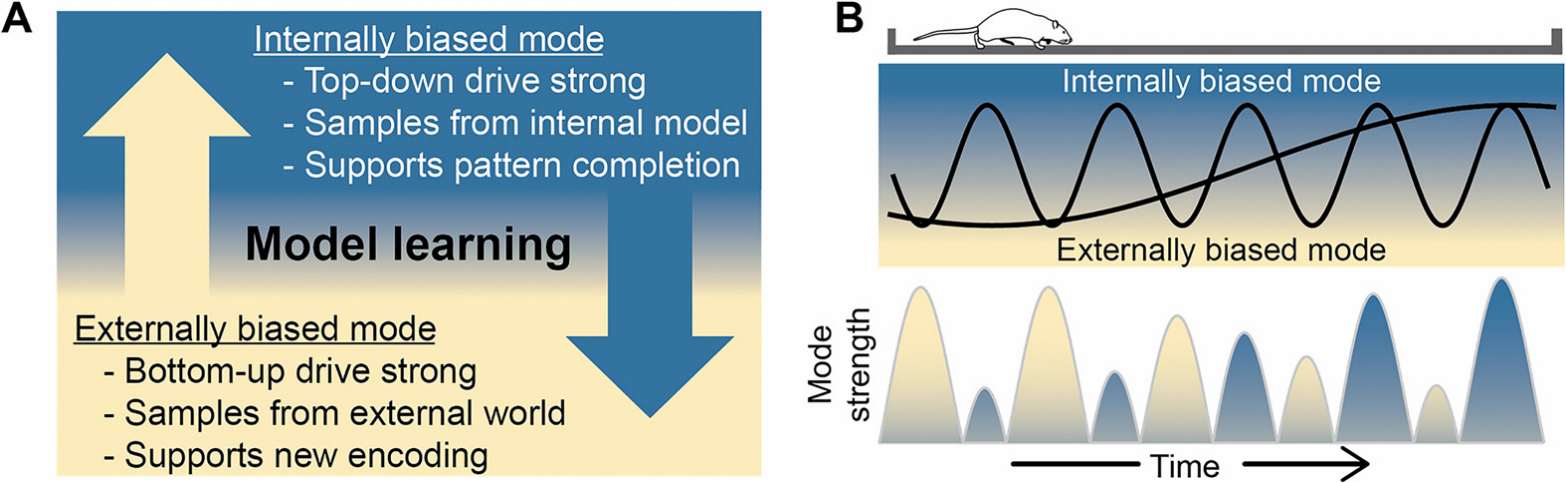
Switching between internally and externally biased modes. (A) Schematic of internally and externally biased modes of processing. (B) Illustration of switching between external and internal drivers of activity at multiple timescales simultaneously. In the time it takes for an animal to navigate a linear track, areas like the hippocampus switch between internally and externally biased modes at fast and slow timescales simultaneously, as caricatured by the two black sine waves. The net result, as shown by the bottom mode strength chart, is that each mode is sampled often but the dominance of one mode over the other changes gradually.

What are the modes between which neural systems vary? Building on ideas developed by Hasselmo (1995), we refer to them as “internally biased” and “externally biased” modes. The basic properties of these modes are summarized in Figure 1. The “internal” mode is biased toward recurrent or top-down drivers. In this mode, prior learning shapes the neural dynamics, effectively driving the current activation state toward the learned patterns that comprise an internal model. Functionally, this supports perceptual processing by enabling pattern completion and prediction. The “external” mode, in contrast, enhances the relative strength of feedforward drive. In this mode, incoming signals from the world or afferent regions shape the neural dynamics, effectively driving the current activation state to reflect the structure of the input.

**Figure 2.**
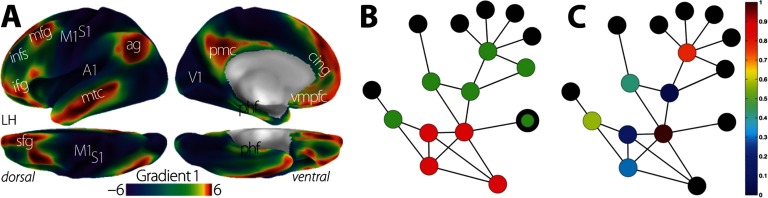
Defining internally and externally biased circuits. (A) Macroscopic gradients from internal to external processing can be defined based on covariation in functional connectivity patterns. Network-theoretic tools such as (B) k-shell decomposition and (C) core-periphery profiling can also be used to define a node-specific measure of distance from the network core. Panel A is adapted from Margulies et al. (2016). Panels B and C are adapted from Della Rossa et al. (2013).

Which anatomical pathways correspond to “internal” and “external” processing? The answer depends on how information flows from the world to arrive at each neural system. In the case of V1, “external” input is provided by the lateral geniculate nucleus, while “internal” input involves local pyramidal neurons and top-down visual projections. In the case of the hippocampus, “external” input is provided via superficial layers of the entorhinal cortex, while “internal” input depends on synapses within and between CA3 and CA1. When considering the cerebrum as a whole, most “external” input is associated with thalamic projections to sensory cortices, while “internal” input occurs most strongly from higher-order cortices and in medial temporal and limbic systems. As a rule of thumb, more “internal” circuits are located a larger number of synapses away from the sensory periphery. Quantitative metrics can also be defined: Figure 2 illustrates a large-scale gradient of external-internal processing derived by Margulies et al. (2016) and network-theoretic tools that can quantify internal-external gradients (Della Rossa, Dercole, & Piccardi, 2013). Most fundamentally, external pathways are those that convey information from current or recent states of the world; internal pathways are those that convey information from a model of the world shaped by days and years of experience.

How might mode-switching aid learning? Computationally, neural systems are believed to learn by changing their representations according to the difference between expected and observed input. Switching between modes that are biased toward new or old information allows neural circuits to identify the contrast between these two sources of information, and thus to iteratively reduce the mismatch between them.

The sections below elaborate the evidence and reasoning behind this proposal. First, we outline the long-standing computational motivation for mode-switching. Then we review the empirical support for mode-switching in both the allocortex and the neocortex on faster timescales (milliseconds and seconds) and slower timescales (minutes to hours). Empirical support for the existence of mode-switching is substantial, while evidence for its role in learning is still in early stages. The theoretical utility of mode-switching leads us to the strong prediction that where there is evidence for switching between internally and externally biased modes, this may be used by the brain to benefit learning. Finally, we consider why the fluctuations occur on such a range of timescales, as well as the broader implications and predictions of this framework.

## COMPUTATIONAL ROLE OF SWITCHING MODES

To think and act adaptively, animals build internal models that reflect relevant properties of the environment. Models of the environment enable diverse behaviors (including navigation, planning, tool use) and mental functions (search in noisy environments, imagery, prediction).

Many algorithms are known to shape accurate internal models in neural networks. The simplest algorithms are Hebbian: connections are strengthened between neurons that represent stimuli that occur together in space and time (Hebb, 1949; Hopfield, 1982). These algorithms become more powerful, however, when the learning process is further driven by a mismatch, or error, signal (Rosenblatt, 1958; Widrow & Hoff, 1960). The mismatch signal can be generated by switching between two modes. In the first mode, the network uses internal representations to generate a “guess” about the present environment (auto-encoder) or near-future environment (predictor). In the second mode, network activity is primarily driven by external input. To generate the mismatch signal, algorithms compare the patterns of activity resulting from these two modes.

The mismatch can be computed at an output layer and then propagated back to earlier layers (e.g., backpropagation; Lillicrap, Cownden, Tweed, & Akerman, 2016; Rumelhart, Hinton & Williams, 1986), or individual units within each layer of a network can compute mismatch based on more local information (e.g., Bengio, Lee, Bornschein, Mesnard, & Lin, 2015; Hinton & McClelland, 1988; O’Reilly, 1996; Smolensky, 1986). Synaptic weights are then iteratively adjusted away from the internal model and toward the external input, such that the next time a similar input is encountered, the network is incrementally better able to anticipate and process it.

We propose that different brain systems, operating on multiple different timescales, switch between internal and external modes as a way of implementing a comparison computation of this kind. Using the mismatch between external measurement and the expectation of the internal model, each neural circuit iteratively improves an internal model of its own “environment,” which is the set of inputs it receives.

## EMPIRICAL EVIDENCE FOR INTERNAL/EXTERNAL MODE SWITCHES

Below we consider a few examples of mode-switching systems (Table 1). This list is representative rather than exhaustive, with the aim of illustrating (a) the range of systems that exhibit functionally relevant switching; (b) the range of timescales over which switching occurs; and (c) the possible neurophysiological commonalities across the examples.

**Table 1.** Summary of internal versus external mode examples.

	**Timescale**	**Spatial scale**	**Internal mode**	**External mode**
**Hippocampal encoding vs. retrieval**	10s ms	Single circuit: hippocampal trisynaptic loop	Trough of CA1pyramidal theta: CA3 input to CA1 stronger than entorhinal input	Peak of CA1pyramidal theta: entorhinal input to CA1 stronger than CA3 input

**Hippocampal encoding vs. retrieval**	100s ms	Multiple circuits: hippocampus and septal circuits	Retrieval: lower cholinergic tone; CA3 drives CA1 activity	Encoding: higher cholinergic tone; entorhinal cortex drives CA1 activity

**Neocortical feedforward vs. feedback**	100s ms to 10s s	Changes often coherent over ∼5–50 mm of neocortex	Field potential filtered in 4–35 Hz range is high and high variance; lower; cholinergic tone; inhibition of core thalamic input and feedforward corticocortical drive	Field potential filtered in 4-35 Hz range is low and low variance; higher cholinergic tone; elevated asynchronous firing, detectable as increase in broadband power

**Active vs. resting wake periods**	seconds–minutes	Most of brain	Lower cholinergic tone; higher overall activity in default mode network	Higher cholinergic tone; lower overall activity in default mode network

**Non-REM vs. REM**	10s minutes	Entire brain	REM: exploration of cortical networks containing long-term memories	Non-REM: dominance of hippocampus, containing more recent memories

**Wake vs. sleep**	Hours	Entire brain	Sleep: minimal influence of environment; relatively lower cholinergic tone in cortex on average	Wake: (potential for) strong influence of environment; relatively higher cholinergic tone in cortex on average

### Hippocampus

The hippocampus provides multiple examples of internal/external mode-switching with demonstrable functional implications. In hippocampal area CA1, “bottom-up” input arrives via a monosynaptic pathway directly from the entorhinal cortex, while “top-down” input arrives from hippocampal area CA3 after traveling the trisynaptic pathway from entorhinal cortex through dentate gyrus to CA1 (van Strien, Cappaert, & Witter, 2009). The bottom-up pathway is more directly influenced by the external world than the trisynaptic pathway, with fewer synapses separation from the environment. Electrophysiological analysis reveals that when animals navigate, CA1 receives input from these two pathways alternately across the phases of the theta rhythm in the local field potential (Brankack, Stewart, & Fox, 1993; Buzsáki, Czopf, Kondákor, & Kellényi, 1986). The alternating phases of CA1 input are functionally dif ferent: CA1 firing represents information immediately in front of the animal during entorhinal bottom-up drive as opposed to retrospective information during CA3 top-down drive (Fernández-Ruiz et al., 2017; Itskov, Pastalkova, Mizuseki, Buzsáki, & Harris, 2008; O’Keefe & Recce, 1993). At a physiological level, these alternating phases also differ with regard to neural plasticity: long-term potentiation (LTP) versus long-term depression (LTD) are induced by activity during the bottom-up and top-down phases, respectively (Huerta & Lisman, 1995; Hyman, Wyble, Goyal, Rossi, & Hasselmo, 2003). Such differences have led these phases to be ascribed distinct functional roles with regard to memory formation (Hasselmo et al., 2002; Norman, Newman, Detre, & Polyn, 2006). The mechanism supporting the rapid switching is a combination of systems-level and cellular-level interactions that maintain the antiphase relationship between these inputs (Buzsáki, 2002).

The internal and external drives to CA1 also switch on a timescale that is an order of magnitude slower than the intra-theta cycle described above (e.g., Figure 1B, with each phase lasting ∼500 ms instead of ∼50 ms). The key observation is that the strength of the bottom-up and top-down inputs varies, not only within each theta cycle, but also across theta cycles (Colgin et al., 2009; Fernández-Ruiz et al., 2017; Schomburg et al., 2014). Once one pathway becomes activated, it remains activated for two to nine consecutive theta cycles (Colgin et al., 2009), and the strength of the two pathways are inversely related such that it is rare to observe both strong on the same theta cycle (Colgin et al., 2009).

Evidence that these slower fluctuations serve functional roles comes from behavioral studies in rodents showing that the bottom-up pathway remains strong during encoding of a novel object or an unfamiliar maze and that the strength of the top-down pathway grows as animals become able to use mnemonic recollection to drive behavior (e.g., Bieri, Bobbitt, & Colgin, 2014; Fernández-Ruiz et al., 2017). Manipulations that interfere with the bottom-up pathway, such as administration of a cholinergic antagonist, shift the balance toward the top-down pathway, and interfere with encoding (Douchamps et al., 2013; Newman, Gillet, Climer & Hasselmo, 2013). Such evidence, in conjunction with evidence from in vitro studies (Hasselmo & Schnell, 1994), suggests that cholinergic modulation plays a central role in mediating the switching between bottom-up and top-down processing in the hippocampus.

Computationally, both fast and slow forms of hippocampal switching reflect an alternation between epochs of “encoding,” where bottom-up activity drives CA1, and epochs of “retrieval,” where CA3 drives CA1 (Hasselmo, 1995; Hasselmo & Schnell, 1994; Meeter, Murre, & Talamini, 2004). Switching between these two inputs may allow CA1 to compare them, supporting alignment of environmental events and internal models (Bittner et al., 2015; Hasselmo et al., 2002). Consistent with the idea that the regular switching of modes is important for learning, blocking theta oscillations via septal inactivation blocks hippocampal dependent learning in rats (Mizumori, Perez, Alvarado, Barnes, & McNaughton, 1990).

### Neocortex

The neocortex provides multiple examples of mode-switching, and these are most often studied in the context of perception rather than learning. The most well-known switch between externally and internally biased modes is the “alpha” oscillation in the occipital neocortex. Electrical potentials recorded from the scalp above the visual cortex oscillate on a timescale of ∼100 ms (Adrian & Matthews, 1934). Decreases in the amplitude of these oscillations are associated with greater engagement with the external visual world. Sensitivity to external visual stimuli appears to be particularly enhanced at the troughs of individual cycles (Busch, Dubois, & VanRullen, 2009; Jensen & Mazaheri, 2010).

More generally across the mammalian neocortex, band-limited oscillations in the electrical potential are observed with timescales typically ranging from ∼30 to 400 ms (Buzáki & Draguhn, 2004; Groppe et al., 2013; Jasper & Penfield, 1949; Keitel & Gross, 2016). Both the amplitude and phase of these oscillations have been linked to switches between external and internal processing modes. The amplitude of oscillations, especially in the range of 6–18 Hz, decreases when local feedforward processing (external mode) is engaged in a particular area (Miller, Honey, Hermes, Rao, & Ojemann, 2014; Pfurtscheller & Da Silva, 1999), while increases in amplitude (and peaks of individual oscillatory cycles) appear to be associated with a more prior-driven, top-down (internal) mode of processing (Bastos et al., 2015; Halgren et al., 2015; Sherman, Kanai, Seth, & VanRullen, 2016; Van Kerkoerle et al., 2014). Such a phasic alternation between stimulus-driven and prior-driven activity has been posited as the basis of perceptual learning in a recent computational model of early visual cortex (O’Reilly, Wyatte, & Rohrlich, 2014).

The variation in feedforward strength need not occur with a fixed rhythmic timescale. Much of the variation in field potentials, and in the amplitude of rhythmic processes, occurs more slowly than 2 Hz, and may not have a tightly constrained timescale (Honey et al., 2012; Leopold, Murayama, & Logothetis, 2003). Even in early visual cortices, slower variations in field potential (Lakatos, Karmos, Mehta, Ulbert, & Schroeder, 2008) and firing rate (Engel et al., 2016) reflect phasic changes in attention and track perceptual sensitivity.

The strength of neocortical feedback and feedforward drive are continuously switching, and this will impact what is perceived and learned. However, experiments that manipulate the strength and frequency of mode switches (keeping other factors constant) would be needed to establish that the alternation of modes is itself driving learning. In addition, we caution against a one-to-one mapping between field potential amplitude and perceptual sensitivity: Field potentials are only an aggregate measure of a system of interwoven lamina, and in some tasks the most accurate inferences about the external world may occur at intermediate levels of feedforward excitability (Linkenkaer-Hansen et al., 2004; McGinley, David, & McCormick, 2015a).

***Common neuromodulatory and oscillatory patterns in hippocampus and neocortex.*** Cholinergic tone is associated with an “external” mode of processing in the hippocampus and in the cerebral cortex (Newman et al., 2013; Newman, Gupta, Climer, Monaghan, & Hasselmo, 2012a). Hippocampally, cholinergic agonists in area CA1 induce a form of pre synaptic inhibition on the “top-down” inputs from area CA3, biasing processing toward the bottom-up inputs from entorhinal cortex (Hasselmo & Schnell, 1994). In the piriform cortex, acetylcholine acts to increase the relative strength of bottom-up input from the olfactory bulb versus top-down input from cortical sources (Hasselmo & Bower, 1992; Linster & Cleland, 2016). In the neocortex, cholinergic modulation facilitates bottom-up processing by potentiating thalamocortical synapses (Disney, Aoki, & Hawken, 2007; Gil, Connors, & Amitai, 1997; Hsieh, Cruikshank & Metherate, 2000). When acetylcholine drives sensory cortex to an external mode, this is reflected in decreased slow (∼2–10 Hz) field oscillations and increased fidelity of sensory coding (Goard & Dan, 2009; Pinto et al., 2013; see also Warburton, Wesnes, Edwards, & Larrad, 1985).

Fluctuations in cholinergic and noradrenergic tone are associated with fluctuations in arousal, as reflected in spontaneous variation in perceptual acuity and bias, and in physiological parameters such as pupil diameter (de Gee et al., 2017; McGinley et al., 2015b). A study of auditory sensory discrimination (McGinley et al., 2015a) demonstrated that false alarm rates were highest in an auditory discrimination task when pupil diameter was largest and slow auditory cortical oscillations were smallest. Thus, the “external mode” for neocortical circuits appears to be associated with increased cholinergic tone (and perhaps noradrenergic tone) as well as with decreases in local oscillatory activity below 30 Hz. Although the highest levels of arousal are not always optimal for perceptual judgment and decision making (de Gee et al., 2017; McGinley et al., 2015a), it seems clear that fluctuations in cholinergic and noradrenergic neuromodulation can bias the relative influence of internal and external sources of information.

### Whole Brain

***Awake rest states and the default mode network.*** Switching between internal and external modes also occurs on longer timescales. We spend as much as half of our awake time in disengaged states characterized by spans of seconds or minutes of reduced attention to our immediate environment (Killingsworth & Gilbert, 2010; Monto, Palva, Voipio, & Palva, 2008; Sadaghiani, Hesselmann, Friston, & Kleinschmidt, 2010). Why do we continually interleave internally oriented processing in daily function? Our framework suggests this interleaving may help to update internal models to reflect recent experience. Indeed, brain activity occurring after stimulus offset is associated with enhanced subsequent memory (Ben-Yakov & Dudai, 2011; Tambini, Ketz, & Davachi, 2010); disengaged time benefits creative problem solving (Baird et al., 2012); and hundreds of experiments have demonstrated that information is better retained when exposure is spaced across time rather than massed (Cepeda, Pasher, Vul, Wixted, & Rohrer, 2006), suggesting that time away from information is important.

Awake rodents also spend substantial time in disengaged states. They replay past and possible future events during hippocampal sharp wave ripples in quiet rest periods (Diba & Buzsáki, 2007; Johnson & Redish, 2007), when sensory neocortex is in a synchronized “internal mode” (McGinley et al., 2015b). Disrupting these ripples disrupts learning (Jadhav, Kemere, German, & Frank 2012). Periods of quiet rest are associated with lower levels of acetylcholine relative to active rest (Marrosu et al., 1995), and hippocampal sharp wave ripples are blocked by stimulation of cholinergic inputs (Vandecasteele et al., 2014). Thus, this internal mode is again associated with lower cholinergic tone.

The so-called default mode network is a set of interconnected regions far from the sensory periphery, including the posterior parietal cortex, anterior medial cortex, and hippocampus (Figure 2A). Although these individual areas, as discussed in the *Hippocampus* section above, can oscillate between relatively internal and external modes of processing, their mean activation is associated with an overall internal mode of processing for the brain. The default mode network is more active when participants are not engaged in a demanding externally oriented task (Mason et al., 2007; Raichle et al., 2001), and is therefore sometimes called a “task negative” network. However, regions of the default mode network can also be reliably recruited byexternal input that demands understanding of semantics (Binder et al., 1999), constructing scenarios (Hassabis & Maguire, 2007), comprehension of narratives or the viewpoint of others (Marset al., 2012; Simony et al., 2016), or imagining the past and future (Buckner & Carroll, 2007; Spreng, Mar, & Kim, 2009). These are all tasks that require use of high-level internal models. Thus, internal processing can be expressed as a persistent and brain-wide state, as during mind-wandering, but it can also be more transiently and locally interwoven in ongoing behavior and cognition in the external world.

***Sleep and wake.*** At an even longer timescale, the transition between sleep and wake states is perhaps the most dramatic example of switching between internal and external processing modes. During sleep, there is minimal processing of immediate external input—neural dynamics are governed almost exclusively by internal interactions.

Sleep benefits recently formed memories (Stickgold, 2013) and aids the integration of new information into existing memory stores (Tamminen, Lambon Ralph, & Lewis, 2017). Because of the virtual absence of real-time environmental input, sleep may be an ideal time to update internal models to better reflect recent information. The “wake-sleep” neural network learning algorithm, which makes use of switches between internally and externally driven modes to improve internally generated representations of the environment, was so named because of this apparent correspondence (Hinton, Dayan, Frey, & Neal, 1995).

***Rapid eye movement (REM) and non-REM sleep.*** Within a night of sleep, there are several alternations between non-REM and REM stages. This switching may also correspond to a relatively internal versus external orientation, even within the sleeping brain. The hippocampus stores traces of the details of recent experiences and replays these experiences during non-REM sleep, often within sharp wave ripples (Nádasdy, Hirase, Czurkó, Csicsvari, & Buzsáki, 1999), which are temporally correlated with spindle events in cortex (Staresina et al., 2015). This replay is thought to help “teach” cortex about these recent experiences, promoting systems consolidation—the transfer of information from hippocampus to cortex (McClelland, McNaughton, & O’Reilly, 1995). In this sense, non-REM sleep approximates an externally driven mode for the neocortex. Although not as externally driven as when those events were actually experienced, it is an opportunity to recap the details of the day’s events, providing additional exposure to information that was recently acquired from the world.

During REM, in contrast, cortical dynamics are less influenced by the hippocampus and driven more by long-established cortical representations (Diekelmann & Born 2010; Hasselmo, 1999). The interleaving of new information during non-REM with old information during REM over the course of the night may then facilitate the integration of new memories into existing cortical networks (Ficca & Salzarulo, 2004; Sara, 2017).

Thus, from the perspective of the cortex, non-REM sleep is an externally oriented mode, using hippocampal retrieval as a proxy for the external world, whereas REM is an internally oriented mode. However, the opposite is true from the viewpoint of the hippocampus. There, low acetylcholine, as during wake, leads to a retrieval-like (internal) mode during non-REM sleep, and high acetylcholine leads to an encoding-like (external) mode during REM sleep (Hasselmo, 1999; Marrosu et al., 1995). These modes promote learning within the sleeping hippocampus as well (Poe, Walsh, & Bjorness, 2010).

In sum, the non-REM/REM cycle may act as a shorter-timescale microcosm of the wake-sleep cycle. In both the transition between sleep and wake and the transition between non-REM and REM, there is a switch between relatively external and internal processing, happening at opposing times for the sleeping hippocampus and cortex, which may drive learning to improve internal models within these circuits.

## CLARIFICATIONS, PREDICTIONS, AND OPEN QUESTIONS

### Could the Brain Achieve Similar Learning Functions Without Switching Between Modes?

Why is it necessary to switch over time between external and internal modes? Could a circuit rather process feedforward and feedback signals simultaneously? Although feedforward and feedback signals almost always commingle, there are two primary advantages to switching their relative efficacy. Firstly, if the same neural circuits simultaneously process external and internal information, then it is difficult for those circuits to separate what is in the environment from what is generated based on priors, memories, and expectations. When newly encoded information is ubiquitously bound to retrieved memories it becomes difficult to distinguish related memories (Hasselmo & Bower, 1993), and reencoding of recently retrieved information can result in runaway synaptic strengthening (Newman, Shay, & Hasselmo, 2012b). Secondly, switching is an important ingredient in algorithms that converge toward accurate high-dimensional internal models (Bengio et al., 2015; Heeger, 2017; O’Reilly, 1996). As noted by Marblestone, Wayne, and Kording (2016), although the specific implementations of the relevant algorithms (recirculation, contrastive Hebbian learning, wake-sleep) may vary, they all invoke “feedback connections that carry error phasically” (p. 6). The general principle is that one should iteratively (a) use one’s best current model of the world to interpret incoming data and (b) use new data to update one’s model of the world. Without switching, it is unclear what data is coming from the environment, and so it is difficult to adjust the internal model to better match that environment. Without switching, it is also difficult to fully instantiate an internal representation when receiving strong environmental input.

What if different neural circuits were specialized for representing internal and external information, thus avoiding the need for alternating modes over time? Although the brain does separate externally biased and internally biased information sources in space (sensory versus higher-order cortical regions, as in Figure 2; granular versus supragranular layers of cortex; possibly even dendrites versus soma, Guergiuev, Lillicrap, & Richards, 2016), this does not obviate the benefits of switching. Because switching provides individual neurons with information from prior and subsequent layers in a neural network, it provides a higher-dimensional learning signal, which is important for learning higher-dimensional models. It is possible for neurons to update their weights using a common scalar error (such as a reward signal) that is generated in a separate neural system, but this form of learning does not solve the credit assignment problem in multilayer systems and is inefficient for high-dimensional representations (Marblestone et al., 2016; Werfel, Xie, & Seung, 2005). One could generate an appropriately high-dimensional learning signal in a separate error circuit if that circuit was of comparable complexity to the circuit being trained, but this would be a very costly approach for wiring, and would require continuous coordination between the trainer and trainee. Instead, a relatively simple and robust approach is to allow circuits to generate error signals locally by switching between consecutive bottom-up and top-down biased activity modes. This switching approach has been adopted not only in machine learning (Box 1) but also in neuronal circuit models of learning (e.g., O’Reilly et al., 2014).

**Box 1. Sufficient ingredients for switch-based learning**

Which of the many fluctuating processes in the nervous system will drive learning? Although it is difficult to say what ingredients are necessary, two general characteristics appear to be sufficient: (a) The fluctuating modes of a circuit should be consecutively biased toward internal and external sources of information; (b) Processes for updating synaptic weights must be sensitive to coactivity of neurons as well as to whether activity occurs in an internally biased or externally biased phase.

(a) Internal and external switching: One phase of a fluctuation needs to be more influenced by feedforward information, while the other phase needs to be more influenced by long-term expectations. The external information is associated with volleys of spiking arriving from the sensory periphery. The internal information is associated with volleys of spiking arriving from higher-order cortical systems, and with patterns of synaptic connections that are stable over days or longer.

(b) Plasticity dependent on activity across switches: A number of algorithms have been proposed for training high-dimensional multilayer models by comparing local activity across and within switches. Contrastive Hebbian learning is a supervised learning approach that can approximate backpropagation in multilayer settings (O’Reilly, 1996; Xie & Seung, 2003; see also Scellier & Bengio, 2017). In contrastive Hebbian learning, the weight update can be thought of as either (a) two separate weight updates with opposite signs during internal and external phases; or (b) a single weight update based on the difference in coactivity during presentation of a stimulus and during presentation of the corresponding target. A more neurobiologically grounded learning process is provided by the XCAL rule (O’Reilly et al., 2014), which updates weights based on the difference in coactivity when the network is near to and far from attractors; switching the gain of feedforward drive can push the system toward and away from such attractors. Finally, Hinton (2007) and Bengio et al. (2015) have argued that spike-timing-dependent plasticity can approximate a weight update similar to backpropagation. Although weight updates are applied in the same way during internal or external phases, switching of the network between feedforward- and feedback-dominated dynamics is still required under these proposals. As Bengio et al. (2015) noted, one of the missing ingredients for a biologically plausible approximation to backpropagation is for computations to be “clocked to alternate between feedforward and backpropagation phases (since the latter needs the formers results)” (p. 1).

Two general observations apply to switch-based learning. First, explicit supervision signals are not a necessary component for switch-based learning in multilayer systems, because each layer can treat its subsequent layer as an activation target. Second, switch-based learning need not be restricted to neurons and synapses, and could also operate at the level of neuronal assemblies that are adjusting their mutual efficacies.

### How Can an Experimentalist Decide Whether a Region is in an Internal or External Mode?

It is important to clarify that internal and external modes are not determined by anatomy but by information flow (Figure 1A). A circuit is pushed toward an external mode when it is influenced by information arriving from the world. A circuit is pushed toward an internal mode when it is influenced by information arriving from an internal model (or set of priors) regarding the world. At any given moment, each circuit is subject to both internal and external influences in some mixture, and their relative strengths will vary (e.g., Figure 1B). We have suggested, as a rule of thumb, that pathways closer to the sensory periphery tend to convey external information, and so when these inputs are dominant then a circuit is in an external mode. However, the flow of information can vary along fixed anatomy (as in the case of cortico-hippocampal interactions during wake and sleep). In addition, fixed anatomy may not have an easily interpretable “bottom up” or “top down” status: Projections to the hippocampus from the nucleus reunions appear to be “bottom up,” as they originate in the thalamus, but because they may convey information from the prefrontal cortex, they could be considered “top down.”

Although the large-scale anatomical architecture is strongly correlated with internal and external information flow (Figure 2A), more difficult cases can be decided via real (or imagined) interventional tests. In particular, the balance between internal and external information can be determined by measuring the effects of (a) changing the world state and (b) changing the content of the internal model. To measure external influence, one might ask the following: If I make a small, transient change in the world (e.g., change the shape of a perceived face), how much would that affect the current activity of the circuit I am measuring? To measure the influence of internal information, one might ask the following: If I make a long-lasting change to the state of the world (e.g., change the shape of all faces that are perceived and learned over years), then how much would that affect the present state of the circuit? The balance of internal and external information can be derived based on which kind of manipulation has the greater effect on the circuit at a particular moment.

### Predictions

(a) The strongest prediction of our framework, for which there is already evidence in some of the discussed examples, is that switching between internally and externally biased modes supports error-driven learning. Therefore, in the case of non-REM/REM cycles, for example, a disruption of the switching should produce a deficit in integrating new hippocampal memories with old information stored in neocortex. Similarly, if the relative contribution of feedforward and feedback influences is altered, this should also impair learning of internal models. For example, if the time in external modes is relatively lengthened, our framework predicts that new information would be encoded but would be less well linked to relevant aspects of the existing model; if time in external modes is shortened, then new experience would not be sufficient to correct errors in the existing model.

(b) If fluctuations between internally and externally biased modes reflect a learning process, then the timescale of fluctuation is a timescale over which error signals are computed. Thus, the error signal that drives learning on subsecond scales (e.g., across hippocampal theta cycles or visual cortical alpha cycles) is computed on subsecond properties of the world, and models subsecond relationships of this input. By contrast, the error signal computed across waking and sleeping states can be influenced not only by transient features of the world, but also by external features that vary over many hours. Thus, sleep may be especially necessary for learning associations between events separated by many minutes or hours.

Within the neocortex, the timescale of fluctuation slows as one moves up the processing hierarchy (Hasson, Chen, & Honey, 2015; Honey et al., 2012). If this fluctuation represents a timescale for comparison that drives learning, then earlier cortical regions should be biased to learn mostly about transient features of their input, while higher-order cortical regions may learn in response to environmental features that change over many seconds or even minutes. This could be tested by presenting a stimulus sequence that contains structure on both short timescales (e.g., phonemes in an artificial language) and long timescales (e.g., probabilistic syntax in an artificial language). The internal/external model can then be tested by manipulating (via pharmacological or electromagnetic bias) the rate of switching between internal and external modes. More rapid switching should lead participants to behaviorally exhibit improved learning of the short timescale structure, relative to the long timescale structure.

More generally, if the extraction of slow features leads to the learning of invariant representations (Wiskott & Sejnowski, 2002), increasingly slow mode fluctuations may bias consecutive stages of processing to learn increasingly invariant representations. Computational modeling indicates that, when learning multiscale structure that is nested in time, a learning system endowed with a hierarchy of slowness can be more powerful (Mozer, 1992) and more efficient (Chung, Ahn, & Bengio, 2016; Schmidhuber, 1992), with higher-order structure being learned by the more slowly varying components of the model.

(c) The learning rate of an individual region might vary inversely according to the speed of its internal/external switching. The learning rate can be thought of as the magnitude of synaptic weight change at each learning opportunity. Learning rates likely vary as one descends the hierarchy from the hippocampus (high learning rate) to medial and inferior temporal regions (medium learning rate) to early visual cortex (slow learning rate; Ahissar & Hochstein, 2004; Norman & O’Reilly, 2003; Yang & Maunsell, 2004; see also Lee & Saxe, 2014).

(d) A final prediction of our model is that in order to drive learning, there should exist a local mechanism in each neural system for comparing predicted states with observed states. If mode-switching occurs on the timescale of seconds, then information about the prior state can be stored in circuit activation (e.g., a slowly changing distributed pattern; Baldassano et al., 2016) and differences may be computed continuously in all phases (Hasselmo et al., 2002; Norman et al., 2006). However, in systems where the internal-external switching occurs more slowly (e.g., over minutes), implementing an update rule such as contrastive Hebbian learning (see Box 1) would pose a greater challenge. There would need to be either (a) a trace that can persist over minutes or longer, to allow for the computation of mismatch between the internal and external phases, or (b) a way to switch the sign of the weight update rule between the internal and external phases. There are many biological and circuit mechanisms that can store single-exposure information across minutes and hours (Barak & Tsodyks, 2014; Benna & Fusi, 2016; Reinartz, Biro, Gal, Giugliano, & Marom, 2014), but it is an important question to consider which of these mechanisms could support computation of the mismatch between past and present.

### Open Questions

Once a mismatch is detected, when are internal models updated to reflect this mismatch? Is there a bias to perform model updating during the internal or external mode? Increased cholinergic and noradrenergic tone is associated with increased stimulus-dependent plasticity in the neocortex and the hippocampus (Gu, 2002). At first glance, this suggests that mismatch-based updates to the internal model may occur preferentially during the “external” mode. However, plasticity is rarely studied in settings where synapses are exposed to interleaved bottom-up and top-down drive or in which neuromodulatory tone and stimulus features are varied dynamically. Thus, it remains an important open question whether the biological instantiation of model updating occurs during one mode, or whether it may depend in a more complex manner on the timing of the switching process.

How does the ongoing switch-based learning we have described relate to reinforcement? Traditionally, reinforcement learning is considered a special case of error-driven learning in which *values*are updated based on the difference between expected and received reward—the reward prediction error (Sutton & Barto, 1998). These differences between expectation and outcome can also be used to build models of the environment, thought to be used by the basal ganglia and medial prefrontal cortex to predict outcomes and select actions (Alexander& Brown, 2011; Daw, Gershman, Seymour, Dayan, & Dolan, 2011). There are clear connections between the principles of adaptive task-related neuromodulation (Yu & Dayan, 2005) and the internal and external switching that we describe here. We have focused on the learn ing of high-dimensional multilevel internal models, a setting in which scalar reinforcement is considered impractically inefficient on its own (Werfel et al., 2005). Nonetheless, the “internally generated” mode-switching processes we have described will be strongly modulated by goal state and reinforcement in real behavior (Box 2). It has even been suggested that basal forebrain cholinergic projections, which modulate both cortical circuit state and plasticity (Sugihara, Chen, & Sur, 2016), may provide a supervisory signal that modulates local sensory learning (Hangya, Ranade, Lorenc, & Kepecs, 2015; see also Poort et al., 2015). Diffuse supervision signals, including those from reward, could greatly accelerate the switch-based learning we have described, and so are an important topic for future developments of our framework.

**Box 2. What drives switches between internal and external modes?**

The transition between internal and external modes can be driven by both exogenous and endogenous factors. Exogenous factors, which come from the sensorium, include rewarding, unexpected, or otherwise salient stimuli. Endogenous factors, which can operate independently of environmental input, include receptor recovery from inactivation, endogenous rhythmicity in neuronal population activity, as well as circadian and homeostatic metabolic processes.

The exogenous and endogenous causes of switching can proceed independently, but in most settings they will be coupled. For example, a salient stimulus can shift or reset the phase of an otherwise endogenous oscillation, and circadian clocks become aligned to the presentation of light and food. Thus, organisms will switch to an external mode when more information is needed from the environment for the current task, and in parallel with this purposive switching, there are also multiple timescales of switching that arise from entrainment of endogenous fluctuations to exogenous cues.

## CONCLUSION

Motivated by the hypothesis that internal-external mode-switching can generically support learning, we have reviewed neural circuits and systems that exhibit such fluctuations. Functionally relevant switching unfolds in the hippocampus and neocortical regions on the scale of milliseconds and seconds, and diffusely across the cerebrum, over minutes and hours within and across wake and sleep. The functional influence of cholinergic tone is similar across these settings and timescales.

The fluctuating processes we reviewed cannot be present only because they serve learning: They arise within a broader context of metabolic dynamics and developmental and evolutionary constraints. However, learning is a fundamental function of nervous systems, and internal models are ubiquitously useful in the brain, because they allow for more efficient representations and robustness to noisy input. Switch-based learning allows internal models to be shaped in a natural and general manner, using only local information. The ingredients of such learning appear to be available in many instances where biological systems oscillate around a set point (Box 1). In this way, each neural circuit, while swimming in the idiosyncratic inputs that compose its world, moves gradually toward a more accurate model of that world.

## ACKNOWLEDGMENTS

The authors thank Aaron Bornstein, Roy Cox, Michael Hasselmo, Kathrin Müsch, Kenneth Norman, and Robert Stickgold for useful comments on earlier versions of this manuscript.

## AUTHOR CONTRIBUTIONS

Christopher J. Honey: Conceptualization; Writing – original draft. Ehren L. Newman: Conceptualization; Writing – original draft. Anna C. Schapiro: Conceptualization; Writing – original draft; Christopher J. Honey, Ehren L. Newman, and Anna C. Schapiro: Writing – review & editing.

## FUNDING INFORMATION

The authors gratefully acknowledge the support of the National Institutes of Health (F32-NS093901 to ACS; MH111439-01 subaward CJH) and the Sloan Foundation (Research Fellowship to CJH).

## TECHNICAL TERMS

Backpropagation: Learning by propagating an output-layer error backward through layers of a network, proportional to how much each connection contributed to the error.
Trisynaptic pathway: A three-synapse pathway for information flow in the hippocampus, from the entorhinal cortex to the dentate gyrus, then on to area CA3, and finally to area CA1.
Hippocampal theta oscillations: Rhythmic fluctuations in the local field potential of the hippocampus, repeating cyclically at 6–10 Hz.
Acetylcholine: An organic chemical that modulates and drives activity in diverse regions of central and peripheral nervous systems.
Sharp wave ripple: Local field potential deflection in the hippocampus, accompanied by 140–250 Hz oscillations.
Wake-sleep algorithm: An unsupervised learning algorithm that iteratively samples bottom-up information (recognition connections, “wake” phase) and top-down information (generative connections, “sleep” phase).
Contrastive Hebbian learning: Learning by adjusting connection weights between neuron *i* and *j* based on the difference in *i* − *j* coactivity across bottom-up and top-down phases.
Granular neocortex: Intermediate layers of the cerebral cortex, which typically contain many granule cells and are often a major recipient of thalamic drive.
Supervised learning: A learning process in which input data are paired with explicit labels or feedback.
